# Exploring the views of key stakeholders on dementia risk prediction in primary care in areas of socioeconomic deprivation: a qualitative study

**DOI:** 10.3399/BJGP.2025.0318

**Published:** 2025-12-16

**Authors:** Rebecca L Morris, Nicola Schmidt-Renfree, Wendy Joseph, David Reeves, Catharine Morgan, Blossom Christa Maree Stephan, Harm van Marwijk, Elizabeth Ford, Sarah Sowden, Lindsey Brown, Eugene Yee Hing Tang

**Affiliations:** 1 University of Manchester, Manchester, UK; 2 Brighton and Sussex Medical School, Brighton and Hove, UK; 3 Population Health Sciences Institute, Newcastle University, Newcastle upon Tyne, UK; 4 Curtin University, Perth, Australia

**Keywords:** dementia, general practice, inequalities, qualitative research

## Abstract

**Background:**

There is growing interest in personalised approaches to dementia risk assessment and prevention. Risk prediction tools can estimate an individual’s likelihood of developing dementia, but none are currently used clinically. Socioeconomic deprivation may reduce opportunities for individuals to engage in healthy behaviours that support brain health even if risk is determined.

**Aim:**

To explore the potential challenges and facilitators in introducing dementia risk prediction tools into UK general practice, with an emphasis on those living in areas of socioeconomic deprivation.

**Design and setting:**

This was a qualitative study set in general practices from three geographical areas of England (Greater Manchester, North East, and South East).

**Method:**

Semi-structured qualitative interviews explored the views of key stakeholders about the use of risk prediction tools for future dementia. These were analysed using thematic analysis.

**Results:**

In total, 71 participants were purposively sampled and interviewed (31 primary care staff; 40 patients). We identified four themes that influenced engagement with dementia risk prediction in general practice: 1) risk as an idea; 2) patient views on the impact of, and choice around, risk screening; 3) embedding risk prediction into clinical consultations; and 4) wider system-level engagement to support adoption of risk prediction tools to prevent dementia.

**Conclusion:**

Dementia risk assessment presents distinct challenges compared with other areas of medicine. Increased public awareness around dementia prevention and risk reduction can facilitate this process. When developing interventions, there needs to be a recognition of the need for a whole-systems approach that supports individuals to adopt strategies to reduce their risk.

## How this fits in

Those living in areas of socioeconomic deprivation are at increased risk of chronic diseases including dementia. Although risk assessment models have been developed to predict an individual’s future risk of dementia, few studies have focused on how to implement dementia risk assessment. This multi-regional qualitative study, which includes views from those living in areas of socioeconomic deprivation, brings together the views of key stakeholders in this process. We outline the key areas that practitioners need to be aware of so that a dementia risk assessment process can be successfully implemented in primary care.

## Introduction

Globally, between 50 and 60 million people are living with dementia and this is set to triple by 2050. In the UK estimates are predicted to increase to nearly 2 million by 2051.^1^ There has been a drive towards finding curative treatments but there is strong evidence around risk reduction strategies, hence the focus globally has also been to reduce incidence through earlier identification and an increase in preventive strategies and interventions.^2^

Early identification of dementia can help individuals and their families to access support and intervention in a timely manner as well as maintain the ability to control decision making and planning about their future care. However, diagnostic rates of dementia remain low compared with the actual numbers of people likely to be living with dementia. Research has consistently found that people’s fear of a future dementia diagnosis is high, and their understanding of modifiable risk factors could be improved.^3^

Several accurate, reliable, and cost-effective electronic dementia prediction tools are available for use in clinical settings such as general practice to assess risk at an individual level.^4^ This has become increasingly important given the emphasis now on modifiable risk factors.^2^ The Lancet Commission has highlighted key modifiable risk factors for dementia such as hearing loss, smoking, obesity, depression, diabetes, and alcohol, all of which are modifiable, particularly within a primary care setting. Furthermore, these factors are often incorporated into risk prediction tools, which aim to predict future dementia across various timescales in not only the general population^4^ but also in at-risk groups such as those who have had a stroke.^5^ An example is the ‘Lifestyle for Brain Health’ (LIBRA) score that utilises modifiable lifestyle-related risk factors to predict incident dementia and cognitive impairment.^6^ A validated and reliable predictive tool is a prerequisite for implementation in daily practice, but this does not mean it will be sufficient to make it acceptable and useful to practitioners and patients.

Research into factors affecting the adoption of clinical decision systems in UK primary care has highlighted a range of considerations that play a role in adoption, such as sensitivity to the patient context, flexibility, ease of control, and non-intrusiveness. Perceived threats to staff autonomy, a lack of clear management guidance, ‘alert fatigue’, and a failure to provide training are also barriers to use.^7^ Conversely, a patient’s decision to take up screening or request for testing will likely be linked to a range of factors including having confidence in their ability to modify their own dementia risk.

Furthermore, there may be additional challenges for those who experience socioeconomic and healthcare inequalities across their lifespan. People experiencing socioeconomic disadvantage are at increased risk of developing chronic diseases such as cardiovascular diseases and diabetes (all of which are risk factors for dementia), with poorer outcomes. Typically, people with a more disadvantaged socioeconomic status may be less able to engage in and enact self-management. Different approaches, therefore, may be needed to reduce, or prevent reinforcing, existing health inequalities.^8–10^ In the context of preventing dementia there is a need to understand the contextual constraints that influence how people may be able to adopt, or not, the advice about reducing their dementia risk.

The purpose of this qualitative study was to explore potential barriers and facilitators relating to the introduction of a dementia risk prediction tool into UK general practice with an emphasis on underserved populations such as those living in areas of socioeconomic deprivation.

## Method

### Study design

We conducted semi-structured interviews with key stakeholders across three areas of England (Greater Manchester, Coastal Sussex, and North East and North Cumbria) between August 2023 until December 2024.

### Patient and public involvement

Patient and public involvement was from the inception of the study and was supported by the first author, who had approached the Primary Care Research in Manchester Engagement Resource (PRIMER) Public Participation Involvement Group at the University of Manchester that includes patients, carers, and members of the public interested in primary care research. Five volunteers who had experience of being a carer for someone living with dementia agreed to be public contributors throughout the research project from the initial application and throughout the research study as members of the public involvement group, with the lay co-chair also being a co-applicant on the funding application (the tenth author).

### Participant sampling and recruitment

We purposefully sampled general practices to capture areas of low and high deprivation from each region. Across the 11 practices, there were comparable proportions of practice Index of Multiple Deprivation (IMD) in the quintiles representing the most deprived (1 and 2) and least deprived areas (4 and 5) (36.4% of practices were in the least deprived areas, *n* = 4) (Table 1), with four practices in each of the North East, North West regions, and three practices in the South East region (Supplementary Table S1).^11^

**Table 1.** Proportion of practices (*N* = 11) by Index of Multiple Deprivation (IMD) score^a^

Practice IMD (quintiles)	Number of practices (%)
1	3 (27.3)
2	2 (18.2)
3	2 (18.2)
4	3 (27.3)
5	1 (9.1)

^a^Quintile 1 is most deprived and 5 is least deprived. Source: Department of Health and Social Care.^11^

### Healthcare professionals

We worked with the local clinical research network to recruit a range of healthcare professional (HCP) stakeholders from each practice. We sought to capture the views of those who would be involved in the process of implementing a dementia risk prediction tool in general practice. We sampled participants with a range of ages, genders, and practice roles.

### Patients

We invited patient participants from the 11 practices we had recruited. Potential patient participants who were either attending their chronic disease annual review or an NHS health check (an annual health check for adults in England aged 40–74 years) were asked if they would be interested in finding out more about the study and were given an information sheet and contact information for the study team. Patient participants were eligible to participate if they were aged between 60 and 79 years of age, and able to offer informed consent to participate. Patients were excluded if they were on the general practice’s register for palliative care, dementia, or mild cognitive impairment. Informed written consent was obtained before the interview, and after participants had read the participant information sheet and invitation letter, and in some cases following a telephone, online, or in-person discussion with a member of the research team.

### Data collection

The interviews were held according to participant preference: in-person; online using Zoom Inc. or Microsoft Teams; by telephone; or via written responses (emailed or hand written). The interview guide explored dementia knowledge and understanding, processes of risk assessment, implementation in practice, and how outcomes should be discussed (see supplementary Information S1). Open-ended questions were organised into different subgroups to explore perception and understanding relating to implementation, ethics, and utility of dementia risk prediction tools in general practice.

All interviews were audio-recorded with explicit permission received in advance and before the interviews commenced. Audio-recordings of interviews were transcribed verbatim using university-approved services or by the researchers (the first, second, or third author). Final sample size was determined by thematic saturation, which was identified when there was a point where no new themes or codes were developed from the analysis between and across patient and HCP participants.^12^ Transcripts were anonymised using pseudonyms and any identifying information was obscured or redacted.

### Data analysis

Data analysis was ongoing throughout the study and followed a thematic approach.^13^ Overall, thematic analysis enables the identification of the essential meaning of lived experience within a phenomenological approach; using an iterative process enables themes to develop as each interview is analysed to identify and interpret key themes. Analysis was led by the first author, a qualitative researcher with experience in primary care research. The research team included qualitative researchers, GPs, and researchers with expertise in dementia and dementia risk prediction tools, along with public contributors. The first three authors immersed themselves in the data by reading the transcripts. Approximately 10% of transcripts were double coded to ensure trustworthiness and rigour. The analysts met regularly to discuss themes and concepts. An initial line-by-line coding was undertaken to create an initial inductive framework from sections of the topic guide, and then refined deductively from the data in consultation with the study team and regular discussions between analysts.

Initial themes were also presented to the patient and public involvement group to get their input and inform subsequent analysis. The data analysis was synthesised into a master dataset organised by themes and sub-themes across all sites to allow comparison of all the themes both within and across sites. Analysis was iterative and the final set of themes and sub-themes were agreed on by all authors.

## Results

In total 71 participants were interviewed; 31 primary care staff (13 GPs, eight practice nurses, two nurse associates, two paramedics, two practice managers, two healthcare assistants, one social prescriber ,and one primary care network coordinator, Table 2) and 40 patient participants. In total, 60% of the patient participants were female (24/40) (Table 3).

**Table 2.** HCP participants by role, gender, and ethnicity (*N* = 31)

Role	Number (%)	Gender, female/male	Ethnicity, *n*
GPs	13 (41.9)	5/8	Asian, 3; British Asian, 3; White British, 6; White other, 1
Nurses	8 (25.8)	7/1	White British, 8
Nurse associate	2 (6.5)	2/0	White British, 2
Paramedics	2 (6.5)	2/0	White British, 2
Social prescribers	1 (3.2)	0/1	White British, 1
Practice managers	2 (6.5)	2/0	White British, 2
PCN coordinator	1 (3.2)	1/0	White British, 1
Healthcare assistants	2 (6.5)	2/0	White British, 2

HCP = healthcare professional. PCN = primary care network.

**Table 3.** Patient participants by gender, age, and ethnicity (*N* = 40)

Gender	Number (%)	Age, years, median (range) mean	Ethnicity
Female	24 (60.0)	69.5 (61–78) 69.2	Asian, 1; White British, 19; White other, 4
Male	16 (40.0)	70 (61–79) 69.4	White British, 15; White other, 1

Overall, participants were supportive of the concept of discussing dementia risk but this initial interest was tempered by a range of factors influencing implementation. Factors affecting the uptake and use of a dementia risk prediction tool to discuss a person’s individual risk related to:

- risk as an idea;
- patient views on the impact of, and choice around, risk screening;
- embedding risk prediction into clinical consultations; and
- wider system-level engagement to support adoption of risk prediction tools to prevent dementia.

These four themes will be discussed in more detail below and data from both patients and HCPs will be presented together to demonstrate the perspectives of all stakeholders related to each theme.

### Risk as an idea

In general, participants tended to express initial support for raising the discussion about use of a dementia risk prediction tool and changes patients could make to reduce their risk of developing dementia. The majority of patient participants were supportive of the idea of discussing their risk of developing dementia and recognised that it fitted with the existing practice of discussing personal risk for other conditions:

*‘I think it would be very good to tell people how they could reduce their chances of developing dementia. Just as they tell us how to reduce the chances of developing high cholesterol …’* (ID257, patient)

Patient participants recognised that knowing their risk was of limited use on its own and support for using a risk prediction tool was caveated with wanting to know what they could do to reduce their chances of developing dementia and to be supported to proactively plan for their future:

*‘Yes. I think it* [a risk prediction tool] *is a good idea, because it gives you the option either to change your lifestyle or, if it is going to be dementia, it gives you time to sort of make sure everything is sorted out before the actual onset … I think it should be optional.’* (ID343, patient)

Similarly from the HCPs’ perspective there was a shared initial enthusiasm for having a discussion about reducing patients’ risk of developing dementia and the links with other vascular conditions that made it amenable to a proactive discussion about risk reduction:

*‘I think it’s got a place in general practice definitely.’* (ID132, HCP)

Many HCPs identified that there was a need to encourage lifestyle changes but recognised that for people from socioeconomically deprived communities this might be a bigger challenge owing to the added costs. Overall, within the participants’ narratives there was considerable overlap in themes across participants irrespective of socioeconomic status or IMD quintile:

*‘If you haven’t got money, or if you haven’t got a car, you can’t go to the big supermarket and buy unprocessed foods. And you might not have a fridge, you might not have a stove. There’s so much inequality. If you have dementia in an area of deprivation, will your outcome be worse compared to more affluent areas? Yes.’* (ID315, GP)

From a patient perspective, there was a similar concern that changes to lifestyle or having the time or money to make lifestyle changes may limit how people can engage with a discussion around reducing dementia risk:

*‘I think financially some people would struggle if they are advised to start eating more healthily, eating better — better food.’* (ID346, patient)

### Patient views on the impact of, and choice around, risk screening

For many participants in the study *‘knowledge is power’* (ID234, practice manager) as it supported them to proactively plan discussions with family members and think of ways they might want to adapt to changes in the future. These views were situated within previous experiences of family members or friends who had lived with dementia. Participants’ narratives were framed so that a discussion about their risk of developing dementia could be empowering to support prevention rather than reactive when there was a diagnosis:

*‘Forewarned is forearmed, I can process it better in my head, if I know what’s happening.’* (ID249, patient)

*‘You can put things into place, can’t you, rather than somebody else doing it for you? … financial things, your care, things like that.’* (ID245, patient)

Conversely, for some patients knowing their risk on its own was considered of limited utility. It was recognised that this was a potentially life-changing discussion that needed to be introduced sensitively and that there will be some people who do not want to know:

*‘It’s a double-edged sword. So, my philosophy is I would like to know what my risk is of developing it … when somebody says to you this is life changing, it could be catastrophic. Not just for the individual but for the family.’* (ID143, patient)

Although there was a recognition by patients that it could be distressing to be told that they were at high risk of developing dementia, a few compared this with being told that they were at high risk of developing cancer (for example, based on family history) and avoiding the discussion meant that it would reduce their opportunity to make informed decisions:

*‘I mean you could be at high risk of cancer but that doesn’t mean to say you are going to get it. But I think it is important to have that and then as I say, you can still make decisions based on that.’* (ID143, patient)

Similarly, participants recognised that potentially being identified as high risk could have a significant impact on their identity, privacy, and control over how their data might be used, and meant there was an explicit need to understand the accuracy of scores being generated:

*‘I genuinely think that some people would be really grateful and other people, if it’s not an inappropriate phrase, might end up “shooting the messenger” … Other people I think would just be like “All right, yeah, can I do something about that please?”’* (ID141, patient)

For patient participants to engage with a discussion of their risk of developing dementia it was important that it was something that they would be able to actively opt-in to:

*‘It’s a big decision to make isn’t it, to take a test like that? So it’s, you know, you’ve got to have time to think about it rather than saying, oh, this is part of your health check.’* (ID253, patient)

The impact of these concerns were particularly exemplified when patients described concerns about obtaining travel or life insurance, and for participants who were working there was a concern about having to disclose their risk to employers, which would have an impact on whether people would choose to opt to know their risk:

*‘…* [they] *might think, well this is going to affect my future job, is it going to have an effect in my working life? Is it going to have an effect on insurance … they might say, no thank you, if it’s going to go on my medical records.’* (ID345, patient)

### Embedding risk prediction into clinical consultations

Introducing risk prediction tools into general practice consultations was seen as important and central to embedding dementia prevention discussions into routine practice. Some GPs reported patients already asking about their risk of developing dementia because of previous experience of family members, friends, or neighbours living with the disease. A risk prediction tool was seen as a useful means to help focus the consultation to a more targeted discussion about modifiable risk factors:

*‘It’s actually about very thoroughly exploring somebody’s fears and hearing that out and then saying, “The reason we have this tool is because we’re going to use this to try and modify your risk now.”’* (ID211, GP)

There were mixed views across patients and practice staff on which HCP should introduce the dementia risk, although participants overall agreed it was about giving patients time and space to ask questions, being sensitive to an individual’s previous experiences with dementia, which was central to how it should be introduced and would be fundamental to how patients might engage with any discussion around dementia risk and prevention:

*‘I think it’s about communication. I think it’s about just explaining … why you're using that process and what it’s for.’* (ID134, HCP)

Starting a discussion about dementia risk assessment was seen as a key aspect of implementation, but consideration was needed regarding the optimal point of care at which to introduce the discussion. Some HCPs thought that starting the discussion in conjunction with the health check (offered to patients ≥40 years old) would be optimal as that could help normalise discussions about healthy ageing as well as potentially be less shocking to the individual. This was considered to be sufficiently early that the patient would not be expected to be at risk of developing dementia soon, hence providing more time to enact lifestyle changes that may be effective at reducing their risk:

*‘It could be either routinely offered like a health check or review, or maybe if it was on the website or social media.’* (ID125, nurse)

Similarly, patients recognised the importance of starting the discussion early, and waiting for when risk prediction tools might have greater predictive accuracy was viewed as limiting their chances of impact on reducing their risk:

*‘The younger the better, because if you can have the healthiest lifestyle you possibly can, from an early age, then surely that’s going to reduce your risk later on. I think it could start at an early age, but perhaps in different formats.’* (ID345, patient)

### Wider system-level engagement to support adoption of risk prediction tools to prevent dementia

Capacity and resources available within the practices were key components identified as influencing how, and if, the additional work could be embedded into routine practice. These were similar across all practices involved in the study, irrespective of their IMD. Financial reimbursement was a key mechanism that would support the adoption of using a risk prediction tool alongside appropriate training and support for practice staff:

*‘… it’s part of QOF* [Quality and Outcomes Framework] *or some financial target or something … frailty was one a while ago where they wanted everyone to have a frailty score and identifying people as moderate, severe frailty, and some funding stream came with that.’* (ID213, GP)

Similarly, the impact on workload because of the sensitivities of explaining patients’ risk of developing dementia was recognised by all the HCPs and there would be a need for a longer time for the consultation that would have a wider impact for practices to be able to take on this additional workload:

*‘Would need a lot of time for the conversation with the results and plans for what could be changed and how to reduce risks.’* (ID315, HCP)

Patients also recognised the limited capacity in general practice and although many preferred to have the initial conversation with a GP they recognised there was a limit in their availability and in practice this was likely to be introduced by a specialist nurse:

*‘I think if it was me I would want to hear it from a doctor because I would think that they are the specialist but in reality I think it would be much more likely that there would be more availability of seeing maybe the specialist nurse.’* (ID141, patient)

One mechanism to support wider engagement and adoption of using a dementia risk tool suggested by practice staff was linking the discussion about dementia prevention with wider vascular health checks to reinforce the importance of overall vascular health and reduce their chances of developing any related condition:

*‘If someone’s been asked to get their risk done it’s a chance to not just assess their dementia, I know we're talking about dementia, but then it’s a chance to do their blood pressure and check their diabetes and their cholesterol and all of that …’* (ID214, GP)

Across HCP interviews there was a recognition that the discussion about prevention of other conditions can be delivered by HCPs in the practice team other than the GP and that they could also have a role in discussing dementia risk as long as they had been trained to deliver the consultation compassionately:

*‘It doesn’t need to be a GP. It needs to be someone delivering the results who has a good understanding of what has been done, and someone who can do it sympathetically.’* (ID315, GP)

The multidisciplinary nature of general practice meant that wider team involvement (for example, social prescribers) was also seen as key in discussions about reducing risk as they could support patients to identify ways to address wider lifestyle changes (for example, support groups, walking groups) that might not require medical intervention (for example, medications) and help reduce the additional workload burden for practice staff:

*‘We’ve got social prescribers in our* [primary care network] *and they would be* [a] *really good, useful resource, to use, to help quide them.’* (ID215, GP)

Alongside this, it was suggested that other practice staff could engage with patients using different platforms, such as social media, to help more widely promote initiatives and raise awareness to foster a sense of collective action:

*‘If there’s apps or tools that we could advertise on, say, our social media account and so on, some of the patients would prefer to use that, and that can be easily linked into their medical records.’* (ID112, GP)

## Discussion

### Summary

In this qualitative interview study with HCPs and patients from a range of GP practices, including from socioeconomically deprived areas, participants were generally positive about the introduction of a future dementia risk assessment into primary care. The study analysis identified four key themes: 1) participants overall supported a discussion of risk as an idea; 2) patient views on the impact of, and choice around, risk screening; 3) embedding risk prediction into clinical consultations; and 4) wider system-level engagement to support adoption of risk prediction tools to prevent dementia.

Some key issues that might influence implementation of such assessments included:

- respect that some patients may not want to participate given the wider implications of a risk assessment, hence ensure that each patient has enough time and information to make an informed decision about opting in to, or out of, the assessment process;
- time the start of the assessment process to also incorporate other aspects of health promotion and disease prevention;
- offer assessments to those from midlife onwards to give individuals more time to make lifestyle changes but also being mindful of those in areas of deprivation where time or financial constraints may limit their ability to adopt suggested lifestyle changes; and
- make use of the primary care team as a whole, including social prescribers, to fully and sensitively support patients in making behavioural and lifestyle changes that can reduce their risk.

### Strengths and limitations

This study has a number of strengths including a diverse sample of key stakeholders from different geographical areas with a focus on areas of deprivation to gain a wide understanding of the different perspectives that might influence the implementation of a dementia risk prediction tool. We do recognise some limitations. First, although efforts were made to purposefully sample patient participants from minority ethnic groups (by asking practices to sample from across their practice demographics and by working with our public contributor group) we were only able to recruit one of the 40 patient participants who was Asian, and all others were White. Second, the sample was deliberately older, that is, between 60 and 79 years of age with a focus on those with chronic diseases or requiring an NHS health check. The views of younger patients, for example, at midlife, were therefore not represented within our data. This may be important, particularly as those living in areas of socioeconomic disadvantage tend to receive diagnoses of chronic disease at a younger age.^14^ Finally, we purposefully sampled practices from both high and low indices of deprivation; however, there was minimal difference in the narratives of patients or practice staff when discussing the issues that might be relevant to them in the implementation of a dementia risk prediction tool apart from issues about being able to afford additional costs to make lifestyle changes.

### Comparison with existing literature

Stigma is often associated with cognitive disorders such as dementia.^15^ Offering risk assessment to individuals, therefore, has to be optional, whether it is offered to the general population or only to high-risk groups such as patients who have had a stroke.^16^ But there also needs to be a shift towards educating the general population around what can be done to reduce the risk of dementia. Fear of dementia is common and stigma often arises because of incorrect beliefs that dementia is a normal part of ageing and that there is nothing that can be done, that in turn obstructs individuals from seeking a diagnosis.^15^ Research has found that even in individuals in high-income settings there is limited knowledge and misconceptions that cognitive impairment and dementia are a part of normal ageing, with limited understanding around risk and protective factors^17,18^ and only limited or superficial knowledge around cause and risk.^19^ International policy is now shifting towards a prevention agenda, with increasing interest around identification of those at the greatest risk of developing dementia.

Positive initiatives, through, for example, the World Health Organization guidelines^20^ on risk reduction of cognitive decline and dementia and the Lancet Commissions,^2,21^ can help promote the positive message that more can be done to prevent dementia. This needs to be reinforced through public health campaigns as well as better education of HCPs, particularly as nearly two-thirds of health and care professionals also incorrectly believe dementia is a normal part of ageing.^15^ This shift in attitude can help to reduce the barriers associated with fear, stigma, and hopelessness to allow patients to find ways to reduce their risk. One concern about this approach, however, is that patients might conflate being told they have a high risk of dementia with a diagnosis of it. Evidence in this study shows that patients can clearly separate out risk versus diagnosis, for example, one participant equated it to being told of a risk of cancer but this not meaning a diagnosis.

Views varied regarding the appropriate point of care at which the topic of dementia risk assessment might best be raised. There are positive reasons to align the risk assessment process with initiatives that promote healthy ageing such as the NHS health check. Starting the discussion in conjunction with the health check could help place dementia risk reduction in the wider context of planning for healthy ageing and addressing risks of other chronic cardiovascular or cerebrovascular diseases, at a time when the patient is still relatively young. The aim of the NHS Long Term Plan is to improve the health of the population and prevent disease in order to reduce health inequalities.^22^ Reducing dementia risk would fit well into these programmes and members of the public have expressed a preference to embed risk assessment within routine health checks.^23^ Many modifiable risk factors for dementia occur in midlife.^2^ It would seem reasonable therefore that individuals should be offered the chance to reduce their risk from midlife onwards, a view expressed by many of the participants in this study. Indeed, when combined into a risk model such as LIBRA, these factors could also be used to identify individuals for primary prevention interventions for dementia, particularly in midlife.^24^ There is also now evidence that a risk-stratified multidomain intervention could improve or maintain cognitive function in the older general population.^25^ However, finding interventions that can target multiple risk factors at once can be challenging, particularly in underserved areas.

### Implications for practice

Risk assessment is often carried out in primary care for other diseases, and is usually accompanied by an associated intervention to reduce risk. Cardiovascular diseases share many of the same determinants. Discussion around developing dementia at any stage is a sensitive topic and often quite challenging for clinicians. Even in the context of a patient already having mild cognitive impairment, clinicians rarely discuss with such patients their risk of developing dementia.^26^ Recommendations have been made to communicate risk in the context of brain health services that include the use of precise and defined risk information, clearly defined time intervals over which the risk occurs, and using plain language.^27^ Such challenging discussions are likely to require more than one single primary care consultation and a trusting relationship with a (single) healthcare provider. To mitigate these challenges, the use of a whole-systems approach in primary care that engages the wider multidisciplinary team might be the most effective way of introducing a dementia risk prediction tool. The practice pharmacist could discuss the importance of medication adherence for other conditions that may increase dementia risk. They could also perform medication reviews to remove medications that may impair cognition, and commence strategies such as lipid-modifying treatment. The social prescriber link workers could discuss healthy eating, weight loss initiatives, and find ways to support the individual in the community. The GP can optimise cardiovascular risk factors and optimise the management of conditions such as diabetes that increase dementia risk.

Overall, dementia risk assessment, particularly in primary care, has an important role in the global movement towards dementia risk reduction. A growing evidence base for modifiable risk factors and accurate and well-validated risk assessment tools, with clear patient involvement throughout the implementation process, are key to this, and our study has identified key enablers and barriers to facilitate this.
